# [2,7-Dihy­droxy-8-(4-phen­oxy­benzo­yl)naphthalen-1-yl](4-phen­oxy­phen­yl)methanone

**DOI:** 10.1107/S1600536812052038

**Published:** 2013-01-09

**Authors:** Daichi Hijikata, Kosuke Sasagawa, Sayaka Yoshiwaka, Akiko Okamoto, Noriyuki Yonezawa

**Affiliations:** a Department of Organic and Polymer Materials Chemistry, Tokyo University of Agriculture & Technology (TUAT), Koganei, Tokyo 184.8588, Japan

## Abstract

In the title compound, C_36_H_24_O_6_, the benzoyl groups at the 1- and 8-positions of the naphthalene system are in an *anti* orientation. Both carbonyl groups form intra­molecular O—H⋯O hydrogen bonds with hy­droxy groups affording six-membered rings. The benzene rings of the benzoyl groups make dihedral angles of 59.26 (13) and 59.09 (13)° with the naphthalene ring system. Zigzag C—H⋯O chains and ladder C—H⋯O chains between the phenoxybenzoyl groups along the *ab* diagonals form an undulating checkered sheet. The molecules are further connected into a three-dimensional network by C—H⋯π interactions.

## Related literature

For electrophilic aromatic aroylation of the naphthalene core, see: Okamoto & Yonezawa (2009 ▶); Okamoto *et al.* (2011 ▶, 2013 ▶). For the structures of (2,7-dimeth­oxy­naphthalene-1,8-di­yl)bis­(4-fluoro­phen­yl)di­meth­anone and 2,7-dimeth­oxy-1,8-bis­(4-phen­oxy­benzo­yl)naphthalene, see: Watanabe *et al.* (2010 ▶) andr Hijikata *et al.* (2010 ▶), respectively.
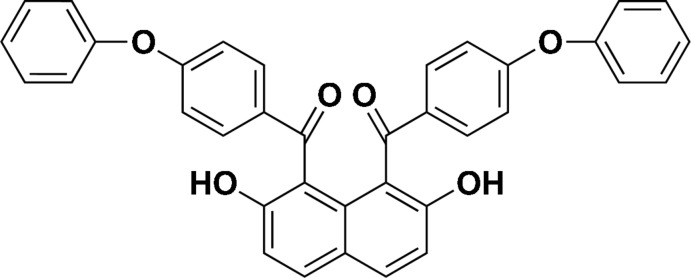


## Experimental

### 

#### Crystal data

C_36_H_24_O_6_
*M* *_r_* = 552.55
Monoclinic, 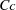

*a* = 16.0313 (3) Å
*b* = 18.4956 (3) Å
*c* = 12.1238 (2) Å
β = 131.389 (1)°
*V* = 2696.95 (9) Å^3^
*Z* = 4
Cu *K*α radiation
μ = 0.75 mm^−1^
*T* = 193 K
0.60 × 0.55 × 0.10 mm

#### Data collection

Rigaku R-AXIS RAPID diffractometer
Absorption correction: numerical (*NUMABS*; Higashi, 1999 ▶) *T*_min_ = 0.661, *T*_max_ = 0.929
22236 measured reflections
4868 independent reflections
4527 reflections with *I* > 2σ(*I*)
*R*_int_ = 0.033

#### Refinement

*R*[*F* ^2^ > 2σ(*F* ^2^)] = 0.037
*wR*(*F* ^2^) = 0.096
*S* = 1.08
4868 reflections
382 parameters
2 restraints
H-atom parameters constrained
Δρ_max_ = 0.20 e Å^−3^
Δρ_min_ = −0.21 e Å^−3^
Absolute structure: Flack (1983 ▶), 2389 Friedel pairs
Flack parameter: 0.05 (19)

### 

Data collection: *PROCESS-AUTO* (Rigaku, 1998 ▶); cell refinement: *PROCESS-AUTO*; data reduction: *PROCESS-AUTO*; program(s) used to solve structure: *Il Milione* (Burla *et al.*, 2007 ▶); program(s) used to refine structure: *SHELXL97* (Sheldrick, 2008 ▶); molecular graphics: *ORTEPIII* (Burnett & Johnson, 1996 ▶); software used to prepare material for publication: *SHELXL97*.

## Supplementary Material

Crystal structure: contains datablock(s) I, global. DOI: 10.1107/S1600536812052038/rn2112sup1.cif

Structure factors: contains datablock(s) I. DOI: 10.1107/S1600536812052038/rn2112Isup2.hkl

Supplementary material file. DOI: 10.1107/S1600536812052038/rn2112Isup3.cml

Additional supplementary materials:  crystallographic information; 3D view; checkCIF report

## Figures and Tables

**Table 1:** Hydrogen-bond geometry (Å, °) *Cg*1 and *Cg*2 are the centroids of the C25—C30 and C31—C36 rings, respectively.

*D*—H⋯*A*	*D*—H	H⋯*A*	*D*⋯*A*	*D*—H⋯*A*
O5—H5*A*⋯O1	0.84	1.83	2.560 (3)	145
O6—H6*A*⋯O2	0.84	1.88	2.563 (3)	138
C26—H26⋯O4^i^	0.95	2.48	3.377 (4)	157
C27—H27⋯O1^i^	0.95	2.51	3.269 (4)	137
C32—H32⋯O3^ii^	0.95	2.49	3.382 (4)	156
C33—H33⋯O2^ii^	0.95	2.51	3.270 (4)	137
C14—H14⋯*Cg*1^iii^	0.95	2.80	3.740 (2)	171
C21—H21⋯*Cg*2^iv^	0.95	2.80	3.740 (2)	171

Symmetry codes: (i) 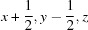; (ii) 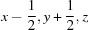; (iii) 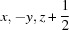; (iv) 
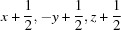
.

**Fig. 1.**
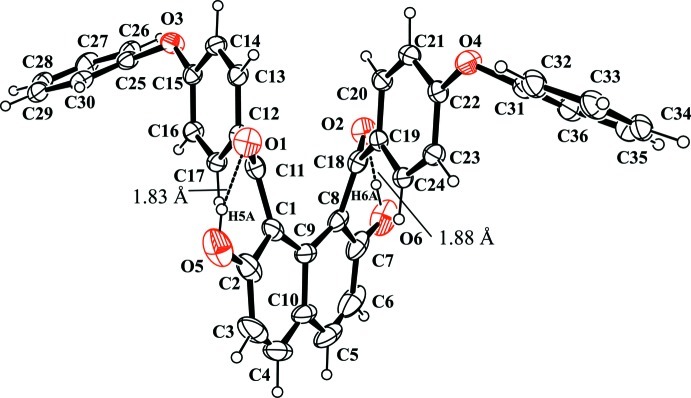
The molecular structure of title compound, showing 30% probability displacement ellipsoids. The intramolecular O—H···O hydrogen bond is shown as a dashed line.

**Fig. 2.**
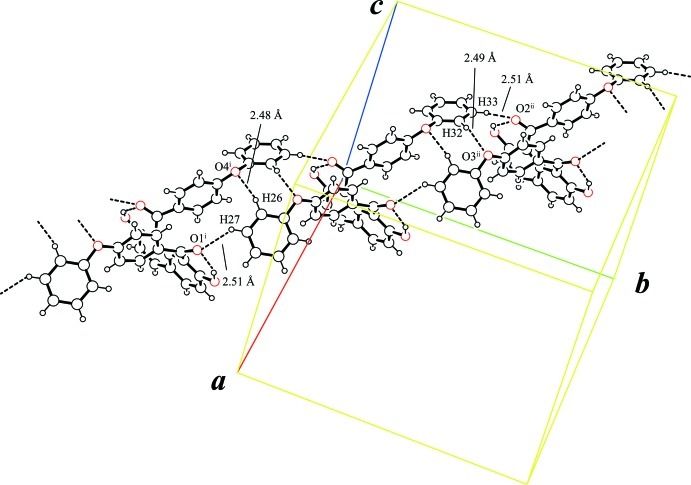
A partial crystal packing diagram of title compound. The intermolecular C—H···O interactions are shown as dashed lines.

**Fig. 3.**
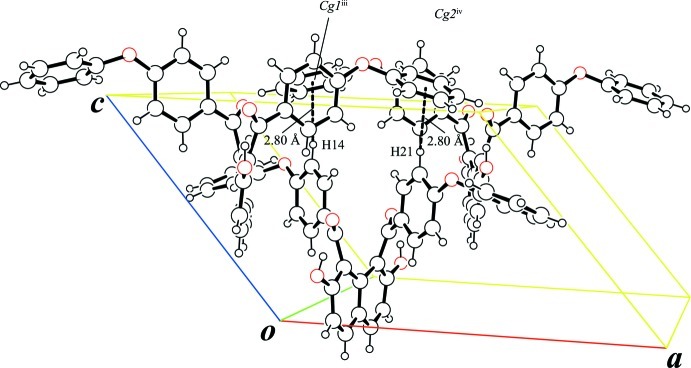
A partial crystal packing diagram of title compound. The intermolecular C—H···π interactions are shown as dashed lines.

## supplementary crystallographic information

### Comment

In the course of our study on electrophilic aromatic aroylation of 2,7-dimethoxynaphthalene, *peri*-aroylnaphthalene compounds have proven to be formed regioselectively with the aid of suitable acidic mediators (Okamoto & Yonezawa, 2009; Okamoto *et al.*, 2011). As one of the applications of *peri*-aroylnaphthalene synthetic studies, the authors have integrated the resulting molecular unit to the poly(ether ketone) backbone *via* nucleophilic aromatic substitution polycondensation (Okamoto *et al.*, 2013). The poly(ether ketone)s composed of 1,8-diaroylenenaphthalene units show unique thermal properties and solubility for organic solvents. These notable properties could arise from the structural features of the 1,8-diaroylene naphthalene units. Under these circumstances, the authors have undertaken the X-ray crystal structural study of several 1,8-diaroylated naphthalene analogues exemplified by (2,7-dimethoxynaphthalene-1,8-diyl)bis(4-fluorophenyl)dimethanone (Watanabe *et al.*, 2010) and 2,7-dimethoxy-1,8-bis(4-phenoxybenzoyl)naphthalene (Hijikata *et al.*, 2010). These molecules have essentially the same non-coplanar features. The two aroyl groups are twisted so they are almost perpendicular to the naphthalene rings.

The molecular structure of the title compound is displayed in Fig. 1. Two benzoyl groups are on the 1,8-positions of the naphthalene ring and are in an *anti* orientation relative to one another. The benzene rings of the benzoyl groups make dihedral angles with the naphthalene ring of 59.26 (13) and 59.09 (13)°, respectively. The dihedral angles between the benzene rings of the benzoyl groups and those of the phenoxy groups are 69.05 (13) and 69.02 (13)°. Both carbonyl groups form intramolecular O—H···O hydrogen bonds with hydroxy groups affording six-membered rings. (Fig. 1, Table 1).

In the crystal structure, the molecular packing of the title compound is stabilized mainly by C—H···O and C—H···π interactions. The aromatic hydrogen atoms of the phenoxy groups form two types of intermolecular C—H···O interactions with the ethereal oxygen atom of the phenoxy groups(C26—H26···O4^i^*=* 2.48 Å, C32—H32···O3^ii^*=* 2.49 Å; Fig. 2 and Table 1) and the carbonyl oxygen atom (C27—H27···O1^i^*=* 2.51 Å, C33—H33···O2^ii^*=* 2.51 Å; Fig. 2 and Table 1). Intermolecular C—H···π interactions between the aromatic hydrogen atom of the benzoyl group and the centroid of the benzene ring of the phenoxy group (C14—H14···*Cg1*^iii^= 2.80 Å, C21—H21···*Cg2*^iv^= 2.80 Å; Fig. 3 and Table 1) are observed.

### Experimental

To a stirring solution of 1,8-bis(4-phenoxybenzoyl)-2,7-dimethoxynaphthalene (1.0 mmol, 580 mg) in dichloromethane (1.0 ml) at 0°C was added 1.0 *M* boron tribromide solution in dichloromethane (4.4 ml) slowly, and the reaction mixture was allowed to reach the room temperature. After the reaction mixture had been stirred at room temperature for 48 h, the reaction mixture was cooled to 0 *^o^*C and very slowly quenched with water and extracted with CHCl_3_. The organic layer thus obtained was dried over anhydrous MgSO_4_. The solvent was removed under reduced pressure to give a cake. The crude product was purified by column chromatography (silica gel, CHCl_3_) to give the title compound (isolated yield 88%). Single crystals suitable for X-ray diffraction were obtained by crystallization from Et_2_O-hexane (*v*/*v* = 1:2).

^1^H NMR δ (300 MHz, CDCl_3_): 6.82–6.84 (4*H*, m), 7.08–7.26 (10*H*, m), 7.40 (4*H*, t, J=7.9 Hz) 7.86 (2*H*, d, J=8.9 Hz), 11.29 (2*H*, s) p.p.m.

^13^C NMR δ (75 MHz, CDCl_3_): 115.13, 117.03, 117.28, 120.02, 122.02, 124.46, 130.00, 130.68, 133.79, 136.09, 155.58, 161.74, 195.80 p.p.m.

IR (KBr): 3396(O—H), 1620 (C=O), 1608, 1583, 1487 (Ar, naphthalene) cm^-1^.

HRMS (*m/z*): [*M* + H]^+^ calcd for C_36_H_25_O_6_, 553.1651 found, 553.1637.

m.p. 464.6–465.9 K.

### Refinement

All the H atoms could be located in difference Fourier maps. All the H atoms were subsequently refined as riding atoms, with O5—H5A = 0.84, O6—H6A = 0.84, C—H = 0.95 (aromatic) Å, *U*_iso_(H) = 1.2*U*_eq_(O) and *U*_iso_(H) = 1.2*U*_eq_(C).

### Crystal data

**Table:**

C_36_H_24_O_6_	*F*(000) = 1152
*M**_r_* = 552.55	*D*_x_ = 1.361 Mg m^−^^3^
Monoclinic, *C**c*	Cu *K*α radiation, λ = 1.54187 Å
Hall symbol: C -2yc	Cell parameters from 14515 reflections
*a* = 16.0313 (3) Å	θ = 4.4–68.1°
*b* = 18.4956 (3) Å	µ = 0.75 mm^−^^1^
*c* = 12.1238 (2) Å	*T* = 193 K
β = 131.389 (1)°	Block, yellow
*V* = 2696.95 (9) Å^3^	0.60 × 0.55 × 0.10 mm
*Z* = 4	

### Data collection

**Table:**

Rigaku R-AXIS RAPID diffractometer	4868 independent reflections
Radiation source: rotating anode	4527 reflections with *I* > 2σ(*I*)
Graphite monochromator	*R*_int_ = 0.033
Detector resolution: 10.000 pixels mm^-1^	θ_max_ = 68.1°, θ_min_ = 4.4°
ω scans	*h* = −19→19
Absorption correction: numerical (*NUMABS*; Higashi, 1999)	*k* = −22→22
*T*_min_ = 0.661, *T*_max_ = 0.929	*l* = −14→14
22236 measured reflections	

### Refinement

**Table:**

Refinement on *F*^2^	Hydrogen site location: inferred from neighbouring sites
Least-squares matrix: full	H-atom parameters constrained
*R*[*F*^2^ > 2σ(*F*^2^)] = 0.037	*w* = 1/[σ^2^(*F*_o_^2^) + (0.0414*P*)^2^ + 1.2872*P*] where *P* = (*F*_o_^2^ + 2*F*_c_^2^)/3
*wR*(*F*^2^) = 0.096	(Δ/σ)_max_ < 0.001
*S* = 1.08	Δρ_max_ = 0.20 e Å^−^^3^
4868 reflections	Δρ_min_ = −0.21 e Å^−^^3^
382 parameters	Extinction correction: *SHELXL97* (Sheldrick, 2008), Fc^*^=kFc[1+0.001xFc^2^λ^3^/sin(2θ)]^-1/4^
2 restraints	Extinction coefficient: 0.00222 (11)
Primary atom site location: structure-invariant direct methods	Absolute structure: Flack (1983), 2389 Friedel pairs
Secondary atom site location: difference Fourier map	Flack parameter: 0.05 (19)

### Special details

**Table:**

Geometry. All e.s.d.'s (except the e.s.d. in the dihedral angle between two l.s. planes) are estimated using the full covariance matrix. The cell e.s.d.'s are taken into account individually in the estimation of e.s.d.'s in distances, angles and torsion angles; correlations between e.s.d.'s in cell parameters are only used when they are defined by crystal symmetry. An approximate (isotropic) treatment of cell e.s.d.'s is used for estimating e.s.d.'s involving l.s. planes.
Refinement. Refinement of *F*^2^ against ALL reflections. The weighted *R*-factor *wR* and goodness of fit *S* are based on *F*^2^, conventional *R*-factors *R* are based on *F*, with *F* set to zero for negative *F*^2^. The threshold expression of *F*^2^ > σ(*F*^2^) is used only for calculating *R*-factors(gt) *etc*. and is not relevant to the choice of reflections for refinement. *R*-factors based on *F*^2^ are statistically about twice as large as those based on *F*, and *R*- factors based on ALL data will be even larger.

### Fractional atomic coordinates and isotropic or equivalent isotropic displacement parameters (Å^2^)

**Table:**

	*x*	*y*	*z*	*U*_iso_*/*U*_eq_	
O1	0.46616 (15)	0.24827 (8)	0.4776 (2)	0.0586 (5)	
O2	0.36810 (15)	0.00189 (8)	0.4778 (2)	0.0589 (5)	
O3	0.78626 (13)	−0.00972 (9)	0.69269 (17)	0.0540 (4)	
O4	0.26329 (13)	0.25987 (9)	0.69275 (17)	0.0540 (4)	
O5	0.3598 (2)	0.31569 (12)	0.2334 (3)	0.0889 (7)	
H5A	0.4028	0.3117	0.3252	0.107*	
O6	0.2301 (2)	−0.06560 (12)	0.2333 (3)	0.0887 (7)	
H6A	0.2881	−0.0652	0.3226	0.106*	
C1	0.33277 (19)	0.18893 (13)	0.2506 (2)	0.0461 (5)	
C2	0.3049 (3)	0.25255 (17)	0.1699 (3)	0.0664 (8)	
C3	0.2213 (3)	0.2516 (3)	0.0165 (4)	0.0985 (15)	
H3	0.2020	0.2950	−0.0374	0.118*	
C4	0.1684 (3)	0.1906 (3)	−0.0546 (3)	0.1016 (16)	
H4	0.1154	0.1909	−0.1591	0.122*	
C5	0.1337 (2)	0.0591 (3)	−0.0547 (3)	0.1027 (16)	
H5	0.0822	0.0587	−0.1592	0.123*	
C6	0.1529 (3)	−0.0033 (3)	0.0189 (4)	0.1012 (15)	
H6	0.1190	−0.0471	−0.0339	0.121*	
C7	0.2216 (2)	−0.00274 (17)	0.1702 (3)	0.0661 (8)	
C8	0.27453 (17)	0.06111 (13)	0.2507 (2)	0.0460 (5)	
C9	0.26698 (17)	0.12502 (15)	0.1775 (2)	0.0487 (5)	
C10	0.1889 (2)	0.1250 (2)	0.0211 (3)	0.0757 (9)	
C11	0.43775 (19)	0.19208 (11)	0.4067 (2)	0.0426 (5)	
C12	0.51884 (16)	0.13161 (11)	0.4748 (2)	0.0369 (4)	
C13	0.59891 (17)	0.12824 (12)	0.6275 (2)	0.0422 (5)	
H13	0.5936	0.1599	0.6843	0.051*	
C14	0.68556 (17)	0.07991 (13)	0.6977 (2)	0.0452 (5)	
H14	0.7375	0.0764	0.8019	0.054*	
C15	0.69610 (16)	0.03646 (11)	0.6145 (2)	0.0399 (4)	
C16	0.61693 (17)	0.03743 (12)	0.4626 (2)	0.0413 (5)	
H16	0.6240	0.0065	0.4066	0.050*	
C17	0.52707 (17)	0.08406 (12)	0.3929 (2)	0.0405 (5)	
H17	0.4707	0.0836	0.2888	0.049*	
C18	0.32565 (17)	0.05816 (11)	0.4067 (2)	0.0427 (5)	
C19	0.31261 (16)	0.11833 (11)	0.4748 (2)	0.0368 (4)	
C20	0.38516 (17)	0.12173 (12)	0.6276 (2)	0.0421 (5)	
H20	0.4471	0.0899	0.6844	0.050*	
C21	0.36894 (18)	0.17017 (13)	0.6976 (2)	0.0458 (5)	
H21	0.4215	0.1738	0.8018	0.055*	
C22	0.27547 (17)	0.21371 (11)	0.6151 (2)	0.0399 (4)	
C23	0.20247 (17)	0.21294 (12)	0.4623 (2)	0.0412 (5)	
H23	0.1397	0.2441	0.4063	0.049*	
C24	0.22251 (17)	0.16599 (12)	0.3928 (2)	0.0402 (5)	
H24	0.1746	0.1662	0.2886	0.048*	
C25	0.83934 (16)	−0.02601 (13)	0.6401 (2)	0.0449 (5)	
C26	0.87387 (19)	−0.09629 (14)	0.6575 (3)	0.0530 (6)	
H26	0.8565	−0.1317	0.6964	0.064*	
C27	0.9345 (2)	−0.11477 (15)	0.6174 (3)	0.0567 (6)	
H27	0.9577	−0.1634	0.6274	0.068*	
C28	0.9614 (2)	−0.06375 (15)	0.5636 (3)	0.0553 (6)	
H28	1.0036	−0.0769	0.5372	0.066*	
C29	0.92693 (19)	0.00719 (15)	0.5477 (3)	0.0532 (6)	
H29	0.9457	0.0428	0.5108	0.064*	
C30	0.86529 (18)	0.02621 (13)	0.5855 (2)	0.0480 (5)	
H30	0.8410	0.0747	0.5740	0.058*	
C31	0.15740 (18)	0.27605 (13)	0.6400 (2)	0.0447 (5)	
C32	0.1402 (2)	0.34624 (14)	0.6574 (3)	0.0536 (6)	
H32	0.1966	0.3815	0.6966	0.064*	
C33	0.0399 (2)	0.36505 (15)	0.6174 (3)	0.0565 (6)	
H33	0.0268	0.4137	0.6274	0.068*	
C34	−0.0414 (2)	0.31361 (15)	0.5632 (3)	0.0553 (6)	
H34	−0.1098	0.3267	0.5370	0.066*	
C35	−0.0229 (2)	0.24338 (15)	0.5473 (3)	0.0536 (6)	
H35	−0.0788	0.2079	0.5097	0.064*	
C36	0.0773 (2)	0.22397 (13)	0.5859 (2)	0.0480 (5)	
H36	0.0902	0.1754	0.5751	0.058*	

### Atomic displacement parameters (Å^2^)

**Table:**

	*U*^11^	*U*^22^	*U*^33^	*U*^12^	*U*^13^	*U*^23^
O1	0.0674 (11)	0.0398 (8)	0.0723 (11)	0.0032 (8)	0.0477 (10)	−0.0045 (8)
O2	0.0679 (12)	0.0406 (9)	0.0727 (11)	0.0090 (8)	0.0484 (10)	0.0052 (8)
O3	0.0489 (9)	0.0693 (11)	0.0468 (8)	0.0229 (8)	0.0329 (8)	0.0118 (7)
O4	0.0441 (9)	0.0691 (11)	0.0456 (8)	0.0075 (8)	0.0284 (7)	−0.0114 (7)
O5	0.1121 (19)	0.0656 (13)	0.1298 (19)	0.0416 (13)	0.0974 (18)	0.0511 (13)
O6	0.0801 (15)	0.0650 (13)	0.1276 (19)	−0.0250 (11)	0.0715 (14)	−0.0503 (13)
C1	0.0473 (12)	0.0584 (14)	0.0462 (12)	0.0211 (10)	0.0368 (11)	0.0146 (10)
C2	0.0748 (18)	0.0779 (19)	0.0782 (18)	0.0407 (15)	0.0640 (17)	0.0389 (15)
C3	0.081 (2)	0.161 (4)	0.080 (2)	0.077 (3)	0.064 (2)	0.080 (3)
C4	0.0517 (18)	0.217 (5)	0.0412 (15)	0.054 (3)	0.0328 (14)	0.038 (2)
C5	0.0345 (14)	0.219 (5)	0.0422 (15)	0.004 (2)	0.0201 (13)	−0.042 (2)
C6	0.0496 (17)	0.167 (4)	0.082 (2)	−0.031 (2)	0.0411 (18)	−0.083 (3)
C7	0.0424 (13)	0.080 (2)	0.0774 (18)	−0.0113 (13)	0.0403 (14)	−0.0387 (15)
C8	0.0302 (10)	0.0581 (14)	0.0470 (12)	0.0028 (9)	0.0244 (9)	−0.0147 (10)
C9	0.0329 (11)	0.0798 (15)	0.0347 (10)	0.0158 (11)	0.0230 (9)	−0.0004 (11)
C10	0.0336 (12)	0.159 (3)	0.0319 (11)	0.0234 (16)	0.0206 (10)	−0.0003 (16)
C11	0.0501 (12)	0.0401 (11)	0.0516 (12)	0.0021 (9)	0.0396 (11)	0.0009 (9)
C12	0.0329 (10)	0.0386 (10)	0.0411 (10)	−0.0010 (8)	0.0252 (9)	−0.0009 (8)
C13	0.0372 (11)	0.0491 (11)	0.0418 (10)	−0.0025 (9)	0.0269 (10)	−0.0094 (9)
C14	0.0351 (11)	0.0609 (13)	0.0352 (10)	0.0035 (9)	0.0213 (9)	−0.0013 (9)
C15	0.0341 (10)	0.0437 (11)	0.0426 (10)	0.0057 (9)	0.0257 (9)	0.0040 (9)
C16	0.0405 (11)	0.0445 (11)	0.0421 (10)	0.0030 (9)	0.0287 (9)	−0.0050 (8)
C17	0.0346 (10)	0.0504 (12)	0.0362 (10)	0.0050 (9)	0.0232 (9)	0.0013 (8)
C18	0.0355 (10)	0.0386 (11)	0.0523 (12)	0.0013 (9)	0.0283 (10)	−0.0003 (9)
C19	0.0377 (10)	0.0384 (10)	0.0410 (10)	0.0008 (8)	0.0289 (9)	0.0017 (8)
C20	0.0385 (11)	0.0489 (11)	0.0418 (10)	0.0101 (9)	0.0278 (9)	0.0092 (9)
C21	0.0400 (11)	0.0627 (13)	0.0346 (10)	0.0052 (10)	0.0247 (9)	0.0016 (9)
C22	0.0392 (11)	0.0460 (11)	0.0404 (10)	0.0016 (9)	0.0289 (9)	−0.0025 (9)
C23	0.0379 (10)	0.0450 (11)	0.0405 (10)	0.0104 (9)	0.0259 (9)	0.0054 (8)
C24	0.0350 (11)	0.0509 (12)	0.0360 (10)	0.0034 (9)	0.0241 (9)	−0.0008 (8)
C25	0.0310 (10)	0.0608 (14)	0.0350 (10)	0.0065 (9)	0.0184 (9)	−0.0036 (9)
C26	0.0390 (12)	0.0598 (14)	0.0540 (13)	0.0056 (10)	0.0281 (11)	−0.0001 (11)
C27	0.0425 (12)	0.0593 (15)	0.0600 (14)	0.0066 (11)	0.0303 (11)	−0.0098 (12)
C28	0.0370 (11)	0.0753 (16)	0.0529 (12)	−0.0033 (11)	0.0294 (11)	−0.0173 (12)
C29	0.0395 (12)	0.0713 (16)	0.0431 (12)	−0.0088 (11)	0.0248 (10)	−0.0100 (11)
C30	0.0368 (11)	0.0516 (13)	0.0411 (11)	0.0009 (9)	0.0196 (10)	−0.0073 (9)
C31	0.0428 (11)	0.0600 (14)	0.0358 (10)	0.0109 (10)	0.0280 (10)	0.0043 (9)
C32	0.0589 (15)	0.0611 (14)	0.0552 (13)	0.0054 (11)	0.0439 (13)	0.0000 (11)
C33	0.0672 (16)	0.0597 (15)	0.0581 (14)	0.0193 (13)	0.0481 (13)	0.0102 (11)
C34	0.0507 (13)	0.0771 (17)	0.0521 (12)	0.0203 (13)	0.0400 (11)	0.0176 (12)
C35	0.0514 (14)	0.0697 (16)	0.0444 (12)	0.0038 (12)	0.0336 (11)	0.0090 (11)
C36	0.0547 (13)	0.0530 (13)	0.0417 (11)	0.0106 (10)	0.0342 (11)	0.0075 (9)

### Geometric parameters (Å, º)

**Table:**

O1—C11	1.228 (3)	C16—H16	0.9500
O2—C18	1.230 (3)	C17—H17	0.9500
O3—C15	1.380 (2)	C18—C19	1.481 (3)
O3—C25	1.392 (3)	C19—C20	1.393 (3)
O4—C22	1.377 (2)	C19—C24	1.397 (3)
O4—C31	1.395 (3)	C20—C21	1.372 (3)
O5—C2	1.355 (4)	C20—H20	0.9500
O5—H5A	0.8400	C21—C22	1.383 (3)
O6—C7	1.348 (4)	C21—H21	0.9500
O6—H6A	0.8400	C22—C23	1.391 (3)
C1—C2	1.401 (3)	C23—C24	1.387 (3)
C1—C9	1.435 (4)	C23—H23	0.9500
C1—C11	1.485 (3)	C24—H24	0.9500
C2—C3	1.399 (5)	C25—C26	1.373 (3)
C3—C4	1.329 (6)	C25—C30	1.382 (3)
C3—H3	0.9500	C26—C27	1.387 (4)
C4—C10	1.423 (6)	C26—H26	0.9500
C4—H4	0.9500	C27—C28	1.370 (4)
C5—C6	1.364 (6)	C27—H27	0.9500
C5—C10	1.428 (6)	C28—C29	1.387 (4)
C5—H5	0.9500	C28—H28	0.9500
C6—C7	1.381 (5)	C29—C30	1.381 (3)
C6—H6	0.9500	C29—H29	0.9500
C7—C8	1.404 (3)	C30—H30	0.9500
C8—C9	1.435 (4)	C31—C32	1.372 (3)
C8—C18	1.484 (3)	C31—C36	1.376 (3)
C9—C10	1.422 (3)	C32—C33	1.383 (4)
C11—C12	1.484 (3)	C32—H32	0.9500
C12—C13	1.392 (3)	C33—C34	1.379 (4)
C12—C17	1.396 (3)	C33—H33	0.9500
C13—C14	1.375 (3)	C34—C35	1.374 (4)
C13—H13	0.9500	C34—H34	0.9500
C14—C15	1.385 (3)	C35—C36	1.390 (3)
C14—H14	0.9500	C35—H35	0.9500
C15—C16	1.383 (3)	C36—H36	0.9500
C16—C17	1.387 (3)		
			
C15—O3—C25	119.93 (16)	O2—C18—C8	120.5 (2)
C22—O4—C31	119.97 (16)	C19—C18—C8	121.35 (18)
C2—O5—H5A	109.5	C20—C19—C24	118.61 (18)
C7—O6—H6A	109.5	C20—C19—C18	118.29 (18)
C2—C1—C9	119.7 (2)	C24—C19—C18	122.58 (18)
C2—C1—C11	115.2 (2)	C21—C20—C19	121.27 (19)
C9—C1—C11	124.8 (2)	C21—C20—H20	119.4
O5—C2—C3	117.3 (3)	C19—C20—H20	119.4
O5—C2—C1	122.7 (3)	C20—C21—C22	119.41 (19)
C3—C2—C1	120.0 (3)	C20—C21—H21	120.3
C4—C3—C2	120.9 (3)	C22—C21—H21	120.3
C4—C3—H3	119.6	O4—C22—C21	116.27 (18)
C2—C3—H3	119.6	O4—C22—C23	122.82 (18)
C3—C4—C10	121.9 (3)	C21—C22—C23	120.80 (18)
C3—C4—H4	119.1	C24—C23—C22	119.10 (19)
C10—C4—H4	119.1	C24—C23—H23	120.4
C6—C5—C10	121.7 (3)	C22—C23—H23	120.4
C6—C5—H5	119.2	C23—C24—C19	120.58 (18)
C10—C5—H5	119.2	C23—C24—H24	119.7
C5—C6—C7	120.0 (3)	C19—C24—H24	119.7
C5—C6—H6	120.0	C26—C25—C30	121.1 (2)
C7—C6—H6	120.0	C26—C25—O3	116.2 (2)
O6—C7—C6	116.1 (3)	C30—C25—O3	122.5 (2)
O6—C7—C8	122.9 (3)	C25—C26—C27	119.0 (2)
C6—C7—C8	121.0 (4)	C25—C26—H26	120.5
C7—C8—C9	119.8 (2)	C27—C26—H26	120.5
C7—C8—C18	115.2 (2)	C28—C27—C26	120.8 (2)
C9—C8—C18	124.7 (2)	C28—C27—H27	119.6
C10—C9—C8	117.7 (3)	C26—C27—H27	119.6
C10—C9—C1	117.6 (3)	C27—C28—C29	119.7 (2)
C8—C9—C1	124.71 (18)	C27—C28—H28	120.2
C9—C10—C4	119.0 (3)	C29—C28—H28	120.2
C9—C10—C5	118.9 (3)	C30—C29—C28	120.2 (2)
C4—C10—C5	122.1 (3)	C30—C29—H29	119.9
O1—C11—C12	117.7 (2)	C28—C29—H29	119.9
O1—C11—C1	120.6 (2)	C29—C30—C25	119.3 (2)
C12—C11—C1	121.13 (18)	C29—C30—H30	120.4
C13—C12—C17	118.70 (18)	C25—C30—H30	120.4
C13—C12—C11	118.15 (18)	C32—C31—C36	121.1 (2)
C17—C12—C11	122.61 (18)	C32—C31—O4	116.1 (2)
C14—C13—C12	121.21 (19)	C36—C31—O4	122.6 (2)
C14—C13—H13	119.4	C31—C32—C33	119.3 (2)
C12—C13—H13	119.4	C31—C32—H32	120.3
C13—C14—C15	119.11 (19)	C33—C32—H32	120.3
C13—C14—H14	120.4	C34—C33—C32	120.4 (2)
C15—C14—H14	120.4	C34—C33—H33	119.8
O3—C15—C16	123.03 (18)	C32—C33—H33	119.8
O3—C15—C14	115.80 (18)	C35—C34—C33	119.7 (2)
C16—C15—C14	121.08 (18)	C35—C34—H34	120.1
C15—C16—C17	119.23 (19)	C33—C34—H34	120.1
C15—C16—H16	120.4	C34—C35—C36	120.3 (2)
C17—C16—H16	120.4	C34—C35—H35	119.8
C16—C17—C12	120.47 (18)	C36—C35—H35	119.8
C16—C17—H17	119.8	C31—C36—C35	119.1 (2)
C12—C17—H17	119.8	C31—C36—H36	120.5
O2—C18—C19	117.5 (2)	C35—C36—H36	120.5
			
C9—C1—C2—O5	176.4 (2)	O3—C15—C16—C17	178.0 (2)
C11—C1—C2—O5	−9.4 (3)	C14—C15—C16—C17	1.6 (3)
C9—C1—C2—C3	−7.1 (3)	C15—C16—C17—C12	2.6 (3)
C11—C1—C2—C3	167.1 (2)	C13—C12—C17—C16	−3.8 (3)
O5—C2—C3—C4	175.7 (3)	C11—C12—C17—C16	167.6 (2)
C1—C2—C3—C4	−1.0 (4)	C7—C8—C18—O2	34.8 (3)
C2—C3—C4—C10	4.1 (5)	C9—C8—C18—O2	−151.6 (2)
C10—C5—C6—C7	4.3 (5)	C7—C8—C18—C19	−135.9 (2)
C5—C6—C7—O6	175.6 (3)	C9—C8—C18—C19	37.8 (3)
C5—C6—C7—C8	−1.4 (4)	O2—C18—C19—C20	26.1 (3)
O6—C7—C8—C9	176.5 (2)	C8—C18—C19—C20	−163.0 (2)
C6—C7—C8—C9	−6.7 (3)	O2—C18—C19—C24	−145.5 (2)
O6—C7—C8—C18	−9.5 (3)	C8—C18—C19—C24	25.5 (3)
C6—C7—C8—C18	167.4 (2)	C24—C19—C20—C21	0.5 (3)
C7—C8—C9—C10	11.6 (3)	C18—C19—C20—C21	−171.4 (2)
C18—C8—C9—C10	−161.8 (2)	C19—C20—C21—C22	3.6 (3)
C7—C8—C9—C1	−168.3 (2)	C31—O4—C22—C21	−146.0 (2)
C18—C8—C9—C1	18.3 (3)	C31—O4—C22—C23	37.7 (3)
C2—C1—C9—C10	11.7 (3)	C20—C21—C22—O4	178.6 (2)
C11—C1—C9—C10	−161.8 (2)	C20—C21—C22—C23	−5.0 (3)
C2—C1—C9—C8	−168.4 (2)	O4—C22—C23—C24	178.3 (2)
C11—C1—C9—C8	18.0 (3)	C21—C22—C23—C24	2.1 (3)
C8—C9—C10—C4	171.4 (2)	C22—C23—C24—C19	2.1 (3)
C1—C9—C10—C4	−8.7 (3)	C20—C19—C24—C23	−3.5 (3)
C8—C9—C10—C5	−8.7 (3)	C18—C19—C24—C23	168.1 (2)
C1—C9—C10—C5	171.2 (2)	C15—O3—C25—C26	−140.9 (2)
C3—C4—C10—C9	0.9 (4)	C15—O3—C25—C30	44.7 (3)
C3—C4—C10—C5	−178.9 (3)	C30—C25—C26—C27	−0.8 (3)
C6—C5—C10—C9	0.9 (4)	O3—C25—C26—C27	−175.3 (2)
C6—C5—C10—C4	−179.2 (3)	C25—C26—C27—C28	1.1 (4)
C2—C1—C11—O1	34.9 (3)	C26—C27—C28—C29	−0.5 (4)
C9—C1—C11—O1	−151.3 (2)	C27—C28—C29—C30	−0.3 (3)
C2—C1—C11—C12	−136.1 (2)	C28—C29—C30—C25	0.5 (3)
C9—C1—C11—C12	37.7 (3)	C26—C25—C30—C29	0.0 (3)
O1—C11—C12—C13	26.0 (3)	O3—C25—C30—C29	174.1 (2)
C1—C11—C12—C13	−162.8 (2)	C22—O4—C31—C32	−141.1 (2)
O1—C11—C12—C17	−145.5 (2)	C22—O4—C31—C36	44.7 (3)
C1—C11—C12—C17	25.8 (3)	C36—C31—C32—C33	−1.2 (3)
C17—C12—C13—C14	0.8 (3)	O4—C31—C32—C33	−175.38 (19)
C11—C12—C13—C14	−171.0 (2)	C31—C32—C33—C34	1.2 (3)
C12—C13—C14—C15	3.3 (3)	C32—C33—C34—C35	−0.7 (4)
C25—O3—C15—C16	37.3 (3)	C33—C34—C35—C36	0.2 (3)
C25—O3—C15—C14	−146.1 (2)	C32—C31—C36—C35	0.6 (3)
C13—C14—C15—O3	178.8 (2)	O4—C31—C36—C35	174.45 (19)
C13—C14—C15—C16	−4.5 (3)	C34—C35—C36—C31	−0.1 (3)

### Hydrogen-bond geometry (Å, º)

Cg1 and Cg2 are the centroids of the C25—C30 and C31—C36 rings, respectively.

**Table:**

*D*—H···*A*	*D*—H	H···*A*	*D*···*A*	*D*—H···*A*
O5—H5*A*···O1	0.84	1.83	2.560 (3)	145
O6—H6*A*···O2	0.84	1.88	2.563 (3)	138
C26—H26···O4^i^	0.95	2.48	3.377 (4)	157
C27—H27···O1^i^	0.95	2.51	3.269 (4)	137
C32—H32···O3^ii^	0.95	2.49	3.382 (4)	156
C33—H33···O2^ii^	0.95	2.51	3.270 (4)	137
C14—H14···*Cg*1^iii^	0.95	2.80	3.740 (2)	171
C21—H21···*Cg*2^iv^	0.95	2.80	3.740 (2)	171

Symmetry codes: (i) *x*+1/2, *y*−1/2, *z*; (ii) *x*−1/2, *y*+1/2, *z*; (iii) *x*, −*y*, *z*+1/2; (iv) *x*+1/2, −*y*+1/2, *z*+1/2.
